# Variation in gait parameters used for objective lameness assessment in sound horses at the trot on the straight line and the lunge

**DOI:** 10.1111/evj.13075

**Published:** 2019-02-12

**Authors:** A. M. Hardeman, F. M. Serra Bragança, J. H. Swagemakers, P. R. van Weeren, L. Roepstorff

**Affiliations:** ^1^ Tierklinik Luesche GmbH Luesche Germany; ^2^ Department of Equine Sciences Faculty of Veterinary Medicine Utrecht University Utrecht the Netherlands; ^3^ Department of Anatomy, Physiology and Biochemistry Swedish University of Agricultural Sciences Uppsala Sweden

**Keywords:** horse, gait analysis, clinical, lameness, kinematics

## Abstract

**Background:**

Objective lameness assessment is gaining more importance in a clinical setting, necessitating availability of reference values.

**Objectives:**

To investigate the between ‐path, ‐trial and ‐day variation, between and within horses, in the locomotion symmetry of horses in regular use that are perceived sound.

**Study design:**

Observational study with replicated measurement sessions.

**Methods:**

Twelve owner‐sound horses were trotted on the straight line and on the lunge. Kinematic data were collected from these horses using 3D optical motion capture. Examinations were repeated on 12 occasions over the study which lasted 42 days in total. For each horse, measurements were grouped as five replicates on the first and second measurement days and two replicates on the third measurement day. Between measurement days 2 and 3, every horse had a break from examination of at least 28 days. Previously described symmetry parameters were calculated: RUD and RDD (Range Up/Down Difference; difference in upward/downward movement between right and left halves of a stride); MinDiff and MaxDiff (difference between the two minima/maxima of the movement); HHDswing and HHDstance (Hip Hike Difference‐swing/‐stance; difference between the upward movement of the tuber coxae during swingphase/stancephase). Data are described by the between‐measurement variation for each parameter. A linear mixed model was used to test for the effect of time, surface and path. Intraclass correlation coefficients (ICC) were calculated to access repeatability.

**Results:**

Mean between‐measurement variation was (MinDiff, MaxDiff, RUD, RDD): 13, 12, 20, 16 mm (head); 4, 3, 6, 4 mm (withers) and 5, 4, 6, 6 mm (pelvis); (HHDswing, HHDstance): 7 and 7 mm. More between‐measurement variation is seen on the first measurement day compared to the second and third measurement days. In general, less variation is seen with increasing number of repetitions. Less between‐measurement variation is seen on hard surface compared to soft surface. More between‐measurement variation is seen on the circle compared to the straight line. Between‐horse variation was clearly larger than within‐horse variation. ICC values for the head, withers and pelvis symmetry parameters were 0.68 (head), 0.76 (withers), 0.85 (pelvis).

**Main limitations:**

Lunge measurements on a hard surface were not performed.

**Conclusions:**

Between‐measurement variation may be substantial, especially in head motion. This should be considered when interpreting clinical data after repeated measurements, as in routine lameness assessments.

## Introduction

Objective gait assessment is gradually becoming a standard procedure during lameness exams in equine clinics worldwide, as it overcomes some of the inherent limitations of subjective gait analysis 1. Whereas the ability of experienced observers to detect (subtle) gait irregularities/asymmetries is well recognised 2, there are known limitations to visual subjective gait assessment. These are mainly related to a possible bias effect when performing regional nerve blocks 3 and to the limitations of the human eye in asymmetry perception 4. These confounding factors ultimately contribute to a low repeatability/agreement in subjective lameness assessments 5.

Commonly used systems for objective lameness assessment are based on measurements of movement symmetry of the vertical (i.e. dorsoventral) displacement of the head, pelvis and sometimes withers at trot 1. More pronounced movement asymmetries are commonly related to orthopaedic pain 1. However, movement asymmetry might also be related to a certain extent with non‐pain‐related causes such as handedness 6 and is, as any biological parameter in living beings, subject to biological variation 7. Discrimination between pathological asymmetry and asymmetry due to non‐pathological reasons is essential for the clinical interpretation of the outcome of objective gait analysis 7, as is knowledge of the biological variation in lameness asymmetry for the correct interpretation of repeated measurements, which are common practice in lameness work‐ups.

During those repeated lameness assessments, horses will not change their movement pattern by more than a certain amount, unless as a result of some intervention (e.g. flexion test, diagnostic analgesia). Although there is no strict universal protocol for lameness assessments in the horse, some guidelines that are commonly adhered to exist 7. The lameness exam commonly starts with a trot‐up on the straight and some circles on a hard and a soft surface. After this, flexion tests may be performed if, often followed by diagnostic analgesia. In general, the lameness examination consists more often than not of repeated observations of the same subject under several different conditions. One of the most important outcomes of the examination is a determination of differences between these repeated observations. To avoid over interpretation, it is therefore of utmost importance to establish the extent of normal biological variation between repeated observations that can be expected in healthy horses.

Previous research found a low degree of between‐day repeatability using motion capture in horses, but the outcome was thought to be influenced by the limitations of the 2D motion capture system that was used 8. In both sound and lame horses, good repeatability was found between subsequent measurements spaced five minutes apart using a body‐mounted accelerometer sensor, for head and sacrum vertical displacement symmetry 9.

The objectives of this study were to describe the magnitude of the between‐measurement variation and repeatability of movement symmetry parameters in horses in regular work and judged sound by their owners. This was studied with intervals of 5 and 10 min, within a day, over consecutive days and over a longer period of a time. We hypothesised that there would be a small proportion of biological variation between repeated measurements in this group of horses and that this variation would be smaller within than between horses.

## Materials and methods

### Horses

Twelve sports horses in regular work (3 geldings and 9 mares) with a body mass range of 450–652 kg (mean 551 kg) and an age range of 5–15 years (mean: 8.3 years) were used in this study. The horses were in regular use and deemed sound by their owner or rider. An experienced equine veterinarian examined the horses and graded them as sound or nearly sound (defined as grades 0 or less than 1 on the 0 to 5 AAEP scale 10), on the day before the first measurement. The judgment was based on a subjective assessment of a straight‐line trot up on a soft surface (hard surface was not available). A detailed description of the population can be found in the Supplementary Item 1.

### Marker placement

**Figure 1:**
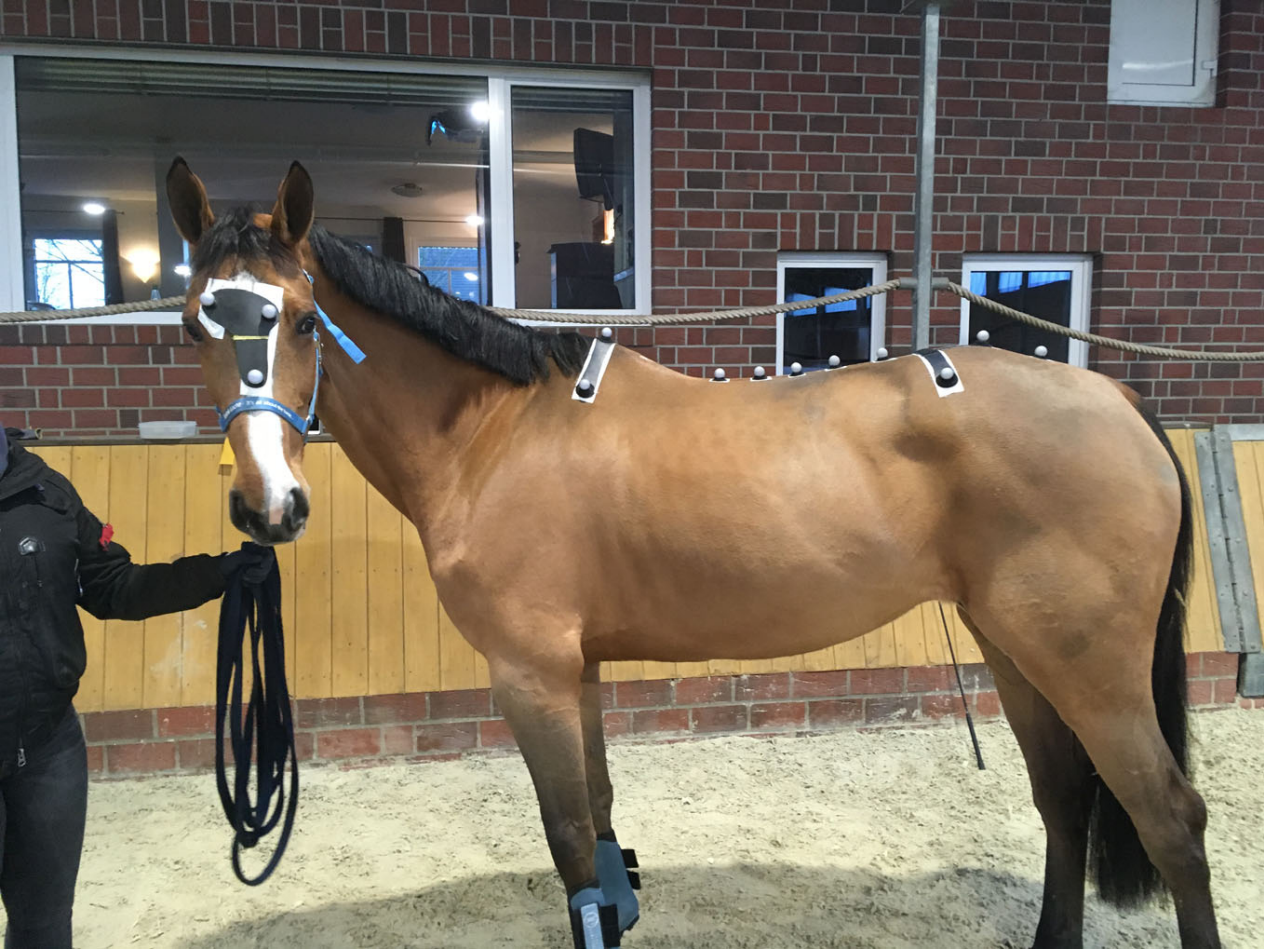
Marker placement in one of the study subjects.

Each subject was equipped with clusters of spherical reflective markers (soft spherical marker, 25 mm diameter^a^), attached to the skin using double‐sided adhesive tape. Three markers were placed in the frontal plane of the head (whereby the lowest marker is used as the reference marker), three markers on the withers (one on the highest point, two markers 20 cm lateral to the central one, one on each side) and three on a T‐shaped strip on respectively the tuber sacrale and the craniodorsal aspect of both tuber coxae (Fig 1). Additional markers were attached to the skin above the dorsal spinous processes of T12, T15, T18, L3, L5 and the sacrum (S5); these were not used in this study. The location of each marker was identified by clipping a small proportion of hair to ensure exact replacement of markers on the following days.

### Data collection

Optical motion capture (OMC) data in 3D were recorded using the Qualisys Motion Capture software (QTM^a^ version: 2.14, build: 3180), connected to 28 high‐speed infrared cameras (Oqus 700+^a^) set to a sampling frequency of 100 Hz. The total covered area in this set‐up was approximately 250 m^2^, height covered was at least 5 m. Calibration before the start of the measurements was done daily according to the manufacturer's instructions. The average calibration residual was 3.2 mm.

### Measuring protocol

**Figure 2:**
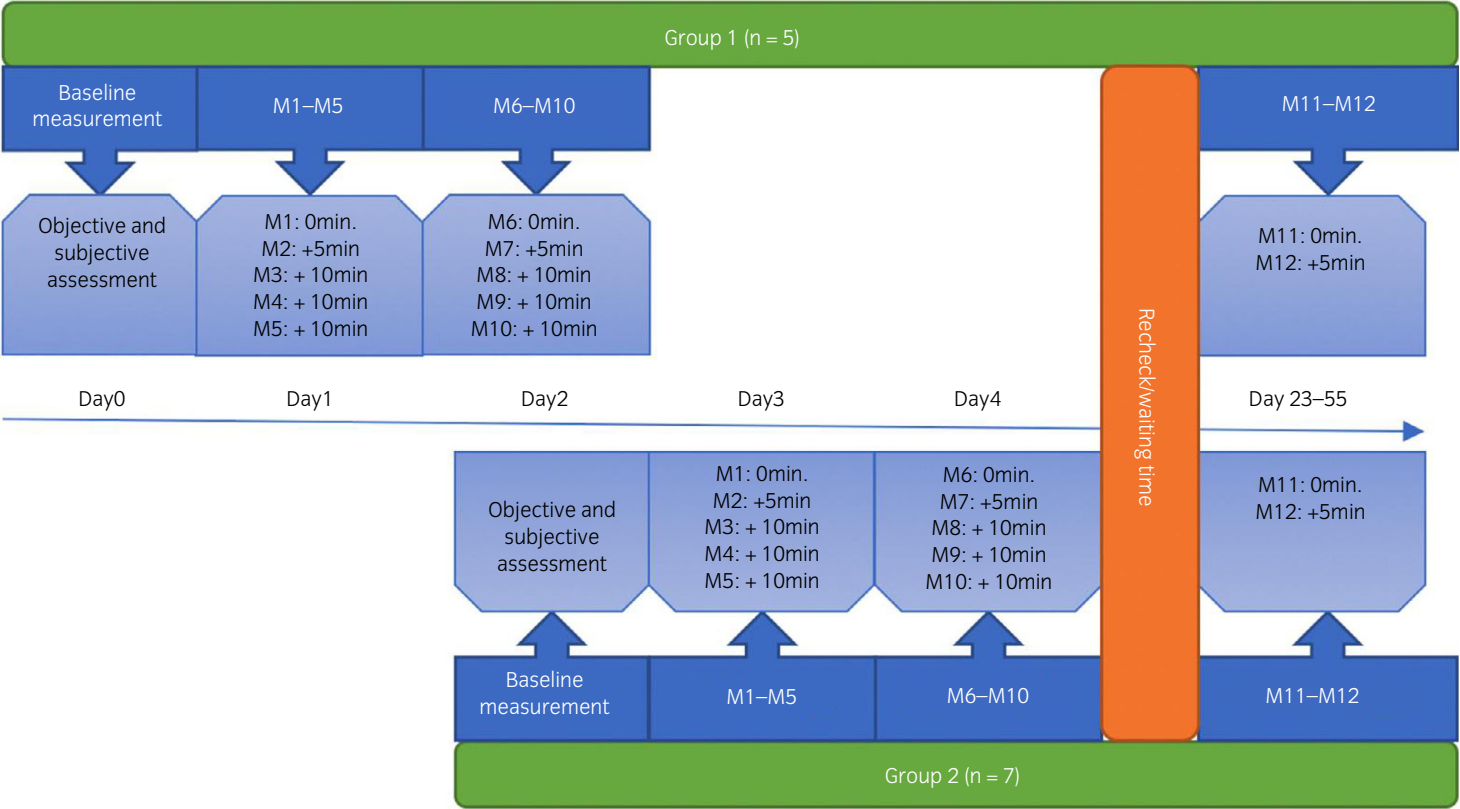
Schematic representation of the study design. More detailed information on the time schedule can be found in Supplementary Item 2. M, measurement.

The horses were divided into two groups (Fig 2). Each group was subjected to the same measuring protocol (details in Supplementary Item 2). Examinations were repeated on 12 occasions over the study which lasted 42 days in total. For each horse, measurements were grouped as five replicates on the first and second measurement days and two replicates on the third measurement day. Between measurement days 2 and 3, every horse had a break from examination of at least 28 days.

Although at different time points, measurements were performed with a 5‐minute interval between the first two measurements of each day (M1‐M2, M6‐M7, M11‐M12) and with 10 minutes in between the remaining measurements of that day (M2‐M3‐M4‐M5, M7‐M8‐M9‐M10) (Fig 2). The same handler always handled all horses in each group.

Each measurement started with a warm‐up period of 5 min hand walking and 10 min lungeing. After the warm‐up up period, markers were placed. Each measurement (M1 to M12) consisted of a hard surface straight line (2 × 20 m) a soft surface straight line (2 × 30 m) and lungeing on a soft surface (diameter approximately 10 m, length of lunge‐line standardised by a knot) on both reins. This protocol is typical for a standard lameness work‐up at the clinic where the study was performed. During the measurements, care was taken to minimise changes in speed, ensuring a steady‐state movement during the whole measurement. Straight line turning was done outside the covered volume.

On the lunge, data were collected for 25 s. The sequence of all measurements (M1‐M12) was hard (tarmac) straight line, soft straight line, left lunge (soft) and right lunge (soft). The soft surface consisted of a combination of sand and synthetic fibre, which was harrowed daily before the first measuring session. Horses were trotted at their preferred speed.

After each measurement, the 3D tracked data were visually inspected ensuring that all markers had been tracked adequately and data were suitable for analysis. Measurements with poor marker tracking or insufficient number of collected strides (five or less complete strides) were discarded. Synchronised video recordings of each measurement were made.

### Kinematic data analysis

Kinematic data were analysed using proprietary software (Qhorse^a^), and several symmetry parameters were calculated, based on the vertical displacement of the different body parts (head, withers, pelvis). These parameters are RUD (Range Up Difference; difference in upward movement between right and left halves of a stride) and RDD (Range Down Difference; difference in downward movement between right and left halves of a stride); MinDiff (difference between the two minima of the movement) 11 and MaxDiff (difference between the two maxima of the movement) 11; HHDswing (Hip Hike Difference‐swing; difference between the upward movement of the tuber coxae during swingphase) 12 and HHDstance (Hip Hike Difference‐stance; difference between the upward movement of the tuber coxae during stance). This gives a total of 14 parameters (Table 1). Parameters are visualised in Supplementary Item 3.

**Table 1:** Between‐measurement variation (in mm), given as the (absolute) prediction interval, per condition and per parameter. Calculated absolute mean variation per (type of) parameter given in the last two columns

	Hard straight	Soft straight	Soft left	Soft right	Mean variation	Mean variation
Head						
MinDiff	12	16	12	13	**13**	**13**
MaxDiff	9	12	11	14	**12**	
RUD	22	20	17	21	**20**	**18**
RDD	15	18	16	14	**16**	
Withers						
MinDiff	3	3	5	4	**4**	**4**
MaxDiff	3	3	3	3	**3**	
RUD	6	5	5	7	**6**	**5**
RDD	4	3	6	4	**4**	
Pelvis						
MinDiff	4	4	5	5	**5**	**5**
MaxDiff	4	4	3	3	**4**	
RUD	6	5	6	6	**6**	**6**
RDD	5	6	6	6	**6**	
HHDsw	7	7	7	7	**7**	**7**
HHDst	6	7	7	6	**7**	

Filtering of the data was done according to earlier investigated methods (F. M. Serra Bragança, unpublished data). A fourth order Butterworth filter with cutoff frequency adjusted to the stride frequency was used. Cutoff frequencies ranged from 1.2 to 1.5 Hz.

### Data analysis

From the data collected, two datasets were generated:

- ‘Non‐offset adjusted’; these are the original data, without any transformations
- ‘Offset adjusted data’; for this data set, each symmetry variable calculated for each horse and each path separately (soft straight, hard straight, soft left and soft right), was offset by subtracting the mean of all measurements from each measurement. This generated a data set centred around zero that allows a better comparison of the between‐measurement variation between horses.

Open software R (3.3.1)^b^ was used for statistical analysis. The package lme4 (version 1.1) was used for the linear mixed effect model and the package merTools (version 0.3.0) for calculating prediction intervals. The linear mixed model analysis was performed on the mean offset adjusted data, for each different path (hard straight, soft straight, soft left, soft right) with the horse ID used as a random effect and repetition used as a fixed effect. For each variable, the prediction intervals were calculated.

To test the effect of time, surface and path, a linear mixed model was used with the offset adjusted between‐measurement variation of each variable (absolute value) as an outcome variable, repetition, day, surface and path as a fixed effect and horse ID as random effect. Normality was checked using q‐q plots and box‐plots and homoscedasticity was checked by plotting the fitted values vs. the residuals. Due to skewness of the residuals, a square root transformation was done and this successfully achieved a normal distribution of the model residuals. Significance was set at P<0.05.

To calculate the repeatability between the different measurements of each parameter, the intra‐class correlation coefficient (ICC) of the non‐offset adjusted data was calculated with the R function ICC.lme (version v 2.2) using the horse ID, surface and path as grouping variables.

## Results

Three horses (horses 3, 8, 10) were not available for the last measuring session (M11‐12). Also, due to a technical problem, one measurement was lost (horse 2, M2, soft left circle).

For the straight‐line trials, the mean (s.d.) of measured strides per trial was 14 (3.8); trotting speed was 3.7 (0.3) m/s. For the lunge trials, the mean (s.d.) of measured strides per trial was 36.8 (5.6); trotting speed was 3.4 (0.2) m/s and circle diameter was 9.7 (0.6) m. The average calibration residuals value was 3.2 mm. The baseline asymmetry (M1) of each horse can be found in Supplementary Item 4. Throughout the study period, none of the horses had a lameness score higher than the chosen threshold of 1/5. Therefore, none of the subjects was excluded from the study.

### Between‐measurement variation

**Figure 3:**
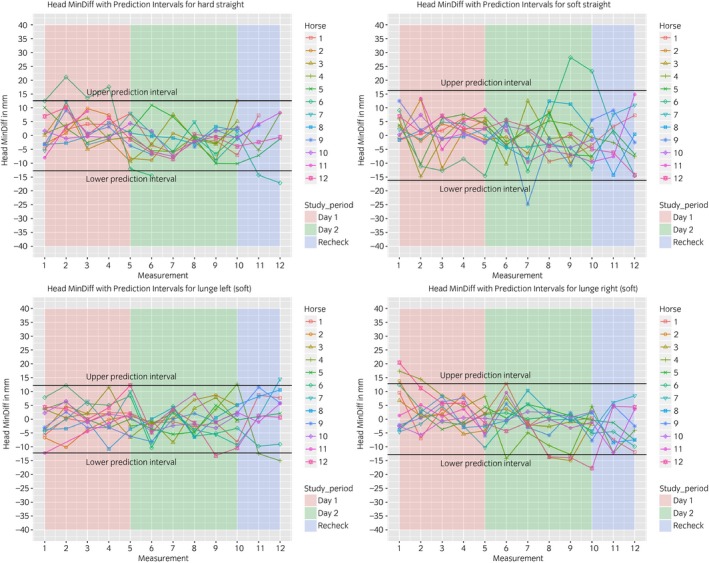
Between‐measurement variation (Offset adjusted data) for ‘MinDiff Head’, per measurement, per day and per horse. Each plot contains one path (hard straight, soft straight, left lunge, right lunge). These data enable the evaluation of the between‐measurement variation between and within horses and between and within days.

**Figure 4:**
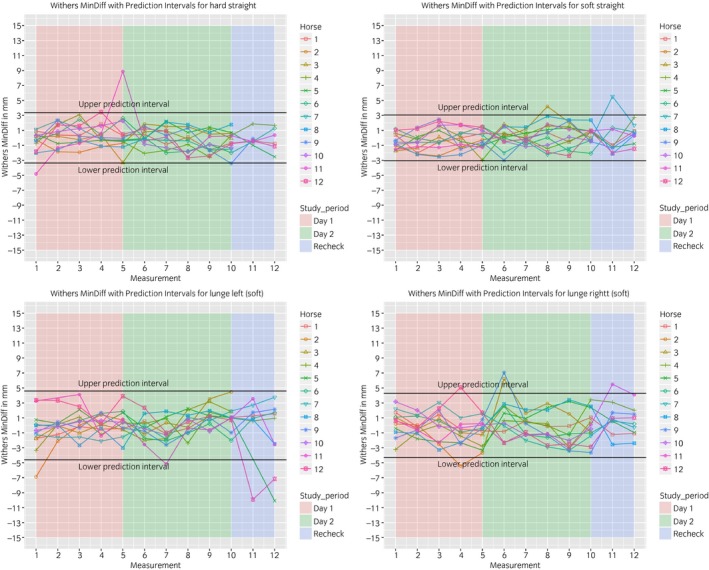
Between‐measurement variation (Offset adjusted data) for ‘MinDiff Withers’, per measurement, per day and per horse. Each plot contains one path (hard straight, soft straight, left lunge, right lunge). These data enable the evaluation of the between‐measurement variation between and within horses and between and within days.

**Figure 5:**
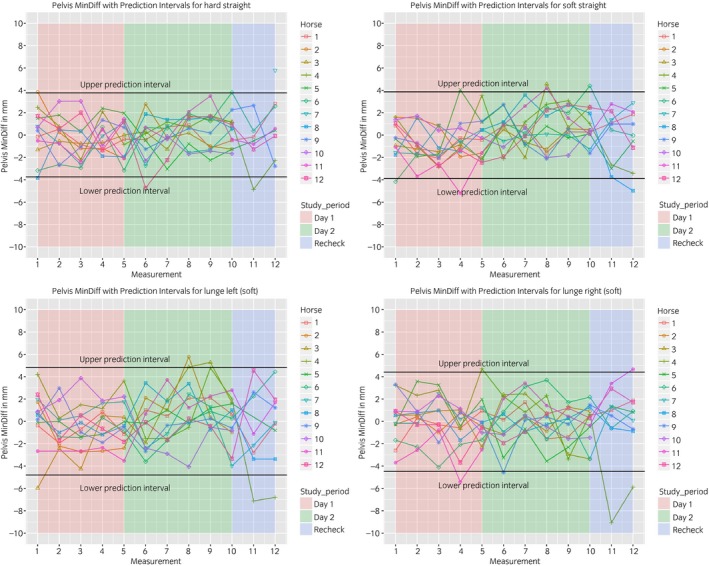
Between‐measurement variation (Offset adjusted data) for ‘MinDiff Pelvis’, per measurement, per day and per horse. Each plot contains one path (hard straight, soft straight, left lunge, right lunge). These data enable the evaluation of the between‐measurement variation between and within horses and between and within days.

**Figure 6:**
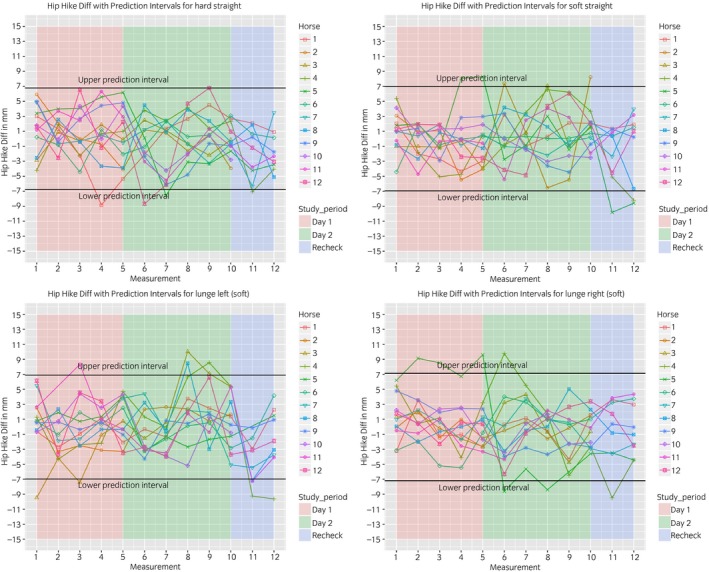
Between‐measurement variation (Offset adjusted data) for ‘Hip Hike Diff’, per measurement, per day and per horse. Each plot contains one path (hard straight, soft straight, left lunge, right lunge). These data enable the evaluation of the between‐measurement variation between and within horses and between and within days.

The between‐measurement variation is visualised in Figures 3, 4, 5, 6. The prediction intervals (Table 1) show the different values of the between‐measurement variation for the various parameters and anatomical locations. Mean between‐measurement variation (over all measurements and over all horses) was: (MinDiff, MaxDiff, RUD, RDD), 13, 12, 20, 16 mm (head); 4, 3, 6, 4 mm (withers) and 5, 4, 6, 6 mm (pelvis); (HHDswing, HHDstance), 7 and 7 mm.

At all anatomical locations, between‐measurement variation of MinDiff and MaxDiff was lower than that of the RUD and RDD (Table 1). The head symmetry parameters showed significantly more variation compared to the withers and pelvis (Table 1, Figures 3, 4, 5, 6).

The mean intra‐class correlation coefficient (ICC) values obtained for the head (0.68) were lower than for the withers (0.76) or pelvis (0.85).

### Effect of time, surface and path on the variation

A reduction of the between‐measurement variation was seen on day 2 and day 3 compared to day 1. In general, there is a tendency to reduced variation with an increasing number of repetitions (for example, M4 and M5 have less variation than M1). This is true for all parameters, but not at all time points.

Less variation was seen on hard surface (straight line) compared to soft surface (straight line).

More variation was seen on the circle compared to the straight line (P<0.01). Detailed model outcomes can be found in the Supplementary Item 5.

### Between vs. within horse variation

Over all measurements, between‐horse variation was substantially higher than within‐horse variation. This becomes graphically evident in the Supplementary Items 6 and 7 as the relatively small individual boxes per horse, compared to the more substantial differences between the different box plots. The observation is also supported by the high ICC values.

## Discussion

This study investigated the amount of ‘between‐measurement variation that can be attributed to biological variation in symmetry parameters over time, over different surfaces and paths (straight line and circles), within and between horses. Knowledge of this variation is paramount for correct interpretation of quantitative gait analysis data in the clinical situation, such as when comparing repeated measurements before and after diagnostic analgesia.

The horses used in this study were assumed to be sound by their owner and in regular training. To clarify, sound in this context is meant as non‐lame. Defining lameness has been discussed before and can also for this study be described as ‘a clinical interpretation of one or more signs indicating a pathological condition of the locomotor system’^13^.

Additionally, the horses were examined subjectively by a clinician and found to have a maximum lameness score of 1/5 on the AAEP scale on the day before the first measurement. Grade 1 of the AAEP scale is described as: ‘Lameness difficult to observe; not consistently apparent regardless of circumstances (e.g. weight carrying, circling, inclines, hard surface).’ It was a deliberate choice to include this scale and not only horses with a grade 0 (‘Lameness not perceptible under any circumstances’), because we wanted to include horses that would be representative of the sports horses population in general (in a recent study 75% of all sports horses in regular work, presumed to be sound by their owners, were subjectively graded as lame by an experienced clinician 14).

For all measured symmetry parameters, the prediction intervals show a certain amount of between‐measurement variation. However, between‐measurement variation is higher for the head parameters compared to those of the pelvis and withers. This is also reflected in the ICC values mentioned above. This difference is in line with previous studies using body mounted accelerometers 9, in which asymmetry parameters of the pelvis on the straight line had better repeatability than head symmetry parameters.

A recent publication showed a lower variation in head parameters compared to our results 21. Possible explanations are the differences in marker placement (our head cluster vs. a single poll sensor) and differences in filtering processes in our motion capture system compared to their sensor‐based system. Our marker placement on the frontal plane introduces extra variation because of the flexion and extension of the atlantico‐occipital joint many horses exhibit when moving. This motion is independent of the displacement of the poll along the Z‐axis, which is the basis for the symmetry parameters.

For all measured anatomical locations, the MinDiff and MaxDiff variation was smaller compared to the RUD and RDD variation, which might be due to the differences in the method used to calculate these symmetry parameters; the MinDiff and MaxDiff consider either the highest or the lowest point of the sinusoidal curves, whereas the RUD and RDD comprise both. This is visualised in the Supplementary Item 3. Variation in the hip‐hike parameters, which also take into consideration the complete range of motion from minima to maxima, is of the same order as the RUD and RDD (Table 1).

These differences in the amount of between‐measurement variation for the different parameters have to be considered, as different objective gait systems use different parameters and one can thus expect different amounts of between‐measurement variation.

We did observe a tendency of increased between‐measurement variation on day 2 and day 3, compared to day 1. Overall, less variation is seen as the repetition increases during a day (for example, M4 and M5 have less variation compared to M1). This is true for all symmetry parameters, but not at all time points. This may be a training effect, although our study objects were accustomed to the measuring environment by a 5‐min hand walk and a 10‐min warming up on the lunge. This training effect has been described earlier on the treadmill 15. For the clinical situation, one should consider this effect and take a, probably horse‐specific, time to let them get familiar to the measuring environment, as these differences in between‐measurement variation might also affect subjective gait evaluation, depending on the magnitude of these differences.

Less variation is seen on hard surface (straight line) compared to soft surface (straight line). We assume this has to do with the more even surface of the hard (tarmac) surface compared to the soft surface (a combination of sand and synthetic fibre). Horses probably need to compensate more for the soft surface, thereby showing more variation in their locomotion pattern and their symmetry parameters.

On the lunge, movement asymmetries are forcibly induced by the circular path 7. Whether the total amount of between‐measurement variation on the lunge would be different compared to the straight had not been investigated yet.

More variation is seen on the circle compared to the straight line. We hypothesise that this might be due to horses experiencing more freedom to change their own speed, circle diameter and body direction on the lunge compared to the straight line. In our circle data, a slight reduction of the average speed over time was observed (comparison not shown), supporting this finding. It might also be more difficult for the handler to perform continuously the same circle speed and diameter between measurements and specially, between days. It is known that these factors affect the degree of measured asymmetry 16.

Nevertheless, the average ICC values for the lunge are higher, indicating less within‐horse variation on the circle compared to the straight line.

The symmetry of both peak vertical displacement and acceleration of the withers is high in sound horses 17. This symmetry was shown to decrease as a result of lameness induction 17. This study demonstrated that the between‐measurement variation of the withers is similar to the pelvis, where a lower between‐measurement variation was found compared to the head parameters (Table 1, Figs 3, 4, 5, 6).

We hypothesise that the higher between‐measurement variation of all head parameters compared to the withers might be a consequence of the relatively high freedom of movement of the head (allowed by the neck) when compared to the withers and pelvis. This high degree of head movement allows the horse to react quickly and with a relatively large amplitude of motion to external stimuli (such as the handler), thus increasing variation in movement symmetry. This difference between anatomical locations has been pointed out earlier in studies with horses in different head and neck positions 18, 19. Some handler effect is inevitable; as the handler will always to a certain extent influence head and neck position and thus head motion. In our study, we tried to minimise this effect as far as possible by letting the horses get accustomed to the test environment and by using the same handler during the entire experiment.

The lower between‐measurement variation of the withers markers compared to the head markers might make it a good candidate aiding in the quantification of forelimb lameness 20.

The substantially higher between‐horse variation compared to the within‐horse variation emphasises the consistency of the locomotion pattern of individual horses, which gives the clinician the obvious possibility to compare the horse with itself over repeated measurements (Supplementary Items 6 and 7).

Previous studies have proposed thresholds to distinguish between sound and lame horses using a sensor‐based system as being 6 mm for the vertical displacement of the head and 3 mm for the pelvis 22, 23. These values are lower than the variation measured with our optical motion capture system. Whereas the use of fixed thresholds for lameness detection can be questioned anyhow 13, the observation underlines that sharply delineated ‘universal thresholds’ can never be established. Differences in outcome are not only influenced by horse‐related factors, but also by system specifications. A previous study concluded that, when comparing two sensor‐based systems, differences in average asymmetry values could be related to differences in sensor hardware, filtering technique, the processing algorithms that derive displacements from the recorded acceleration signals, and the stride detection technique 21. When using optical motion capture, there are comparable technical factors possible that affect the outcome. Standardisation can help here. A recent study showed that, when similar filtering and data processing techniques are applied to both optical motion capture and sensor‐based systems synchronously, a good agreement between the two can be found 24.

It should be kept in mind that, when measuring horses on different locations and at different time points, the practical measurement conditions (i.e. the environment) can also influence outcome and thereby the variation, like the technical aspects alluded to above. These environmental factors include effects on the head and neck position of the horse (by the influence of the handler) 18, 19, surface, the demeanour of the horse 25, circle size 16, speed 6, 16 and probably warming‐up. Besides, one could expect more between‐stride variation in the MinDiff and Maxdiff if the surface is uneven, for example.

When taking all these factors into account, one could still have a poor quality measurement due to unexpected influences (anxious horse, noise from outside). The authors would recommend repeating such measurements or increase the number of collected strides. In that way, variability can be limited to a maximal extent, which is preferable to handling a high stride‐to‐stride variability with a forcibly less accurate mean trial value and high standard deviation. Use of such data can be compared to interpreting bad quality radiographs.

Clinicians should be aware of all these potential influences and outcomes when performing objective gait analysis and/or interpreting data captured elsewhere.

This study has several limitations. The study was performed on a small population of sports horses. Due to limitations of the study location, horses were before inclusion only evaluated on the straight on a soft surface, which is uncommon in clinical practice. Hard surface circles were not included which are nevertheless commonly performed in the clinical situation.

## Conclusion

This study provides data on the variation between measurements in symmetry parameters which can be introduced by the fact that in orthopaedic work‐ups, repeated measuring under different conditions and at different moments is common practice. Repeated measuring was performed at different time intervals on the same day, on consecutive days and after a longer time interval. Measurements were performed on hard and soft surface, on the straight line and on the lunge.

More variation was seen on the first day compared to the second and third measurement day. In general, less variation was seen with increasing number of repetitions. Also, less variation was seen on hard surface (straight line) compared to soft surface (straight line) and more variation on the circle compared to the straight line. The variation within a horse was significantly smaller than the variation between horses.

The results are important for all use of quantitative gait analysis systems and ideally similar studies should be performed with other systems, as this information is a prerequisite for the proper clinical interpretation of data from this type of equipment.

## Authors’ declaration of interests

No competing interests have been declared.

## Ethical animal research

Research ethics committee oversight not required by this journal: noninvasive observational study.

## Owner informed consent

Owners gave consent for their animals’ inclusion in the study.

## Sources of funding

None.

## Authorship

A.M. Hardeman and F. Serra Bragança contributed to planning the experiment, data processing, statistics and preparation of the manuscript. L. Roepstorff contributed to planning the experiment, data collection and preparation of the manuscript. J. Hein Swagemakers and R. van Weeren contributed to planning of the experiment and preparation of the manuscript. All authors have approved the final version of the manuscript.

## Supporting information


**Supplementary Item 1:** Description of the horses used in the study.

**Supplementary Item 2:** Time schedule of all measurements.

**Supplementary Item 3:** Visualisation of the symmetry parameters.

**Supplementary Item 4:** Baseline asymmetry (M1) of each horse.

**Supplementary Item 5:** Model estimates square root transformed for the between measurement ‘absolute deviation’.

**Supplementary Item 6:** Between‐measurement‐variation (non offset data) for MinDiff head, withers and pelvis.

**Supplementary Item 7:** Between‐measurement‐variation (offset data) for MinDiff head, withers and pelvis.

## Manufacturers' addresses

1 Qualisys AB, Motion Capture Systems, Göteborg, Sweden.

2 R‐Studio, Boston, Massachusetts, USA.
