# Supplementary material for: Variation in gait parameters used for objective lameness assessment in sound horses at the trot on the straight line and the lunge
Source: Equine Vet J. 2019 Feb 12;51(6):831–9. doi: 10.1111/evj.13075 (PMC6850282; doi:10.1111/evj.13075)
Supplement: Supplementary file 1 — Supplementary Item 1: Description of the horses used in the study. [file EVJ-51-831-s001.pdf]

BEVA Equine Veterinary Journal

Supplementary Item 1: Details of the study population.

	Year of birth	Gender	Breed	Body mass (kg)	Discipline
**1**	2007	Mare	Baden-Württemberg	450	Jumping/Dressage A
**2**	2011	Gelding	Frisian	553	No competition
**3**	2009	Mare	Hannoveraner	569	Jumping/Dressage A/L
**4**	2012	Mare	Oldenburger	536	Dressage A
**5**	2007	Gelding	KWPN	572	Dressage M/S
**6**	2007	Mare	Westfalen	570	Jumping S
**7**	2011	Mare	KWPN	560	Jumping L
**8**	2012	Mare	Holsteiner	532	Jumping A/L
**9**	2002	Gelding	Holsteiner	652	No competition
**10**	2008	Mare	Hanoveraner	510	Jumping L
**11**	2011	Mare	British sporthorse	498	Jumping L
**12**	2007	Mare	Irish sporthorse	608	Jumping A/L
