# Supplementary material for: Variation in gait parameters used for objective lameness assessment in sound horses at the trot on the straight line and the lunge
Source: Equine Vet J. 2019 Feb 12;51(6):831–9. doi: 10.1111/evj.13075 (PMC6850282; doi:10.1111/evj.13075)
Supplement: Supplementary file 2 — Supplementary Item 2: Time schedule of all measurements. [file EVJ-51-831-s002.pdf]

BEVA Equine Veterinary Journal

Supplementary Item 2: Time schedule of all measurements (M1-M12).

Day	Horse	M1&M6	M2&M7	M3&M8	M4&M9	M5&M10	M11	M12
**1**	1	8:00	8:05	8:15	8:25	8:35		
	2	8:50	8:55	9:05	9:15	9:25		
	3	9:40	9:45	9:55	10:05	10:15		
	4	10:30	10:35	10:45	10:55	11:05		
	5	11:20	11:25	11:35	11:45	11:55		
**2**	1	8:00	8:05	8:15	8:25	8:35		
	2	8:50	8:55	9:05	9:15	9:25		
	3	9:40	9:45	9:55	10:05	10:15		
	4	10:30	10:35	10:45	10:55	11:05		
	5	11:20	11:25	11:35	11:45	11:55		
**3**	6	8:00	8:05	8:15	8:25	8:35		
	7	8:50	8:55	9:05	9:15	9:25		
	8	9:40	9:45	9:55	10:05	10:15		
	9	10:30	10:35	10:45	10:55	11:05		
	10	11:20	11:25	11:35	11:45	11:55		
	11	12:10	12:15	12:25	12:35	12:45		
	12	13:00	13:05	13:15	13:25	13:35		
**4**	6	8:00	8:05	8:15	8:25	8:35		
	7	8:50	8:55	9:05	9:15	9:25		
	8	9:40	9:45	9:55	10:05	10:15		
	9	10:30	10:35	10:45	10:55	11:05		
	10	11:20	11:25	11:35	11:45	11:55		
	11	12:10	12:15	12:25	12:35	12:45		
	12	13:00	13:05	13:15	13:25	13:35		
**28-42**	1-12						0*	+5 min.**

*Horses were measured at different timepoints during the last day (M11).
** M12 was done 5 minutes after M11
