# Supplementary material for: Variation in gait parameters used for objective lameness assessment in sound horses at the trot on the straight line and the lunge
Source: Equine Vet J. 2019 Feb 12;51(6):831–9. doi: 10.1111/evj.13075 (PMC6850282; doi:10.1111/evj.13075)

BEVA Equine Veterinary Journal

Supplementary Item 3: Visualisation of the symmetry parameters. RUD (Range Up Difference; Range Up 1 - Range Up 2); RDD (Range Down Difference; Range Down 1 - Range Down 2); MinDiff (difference between the two minima of the movement); MaxDiff (difference between the two maxima of the movement).

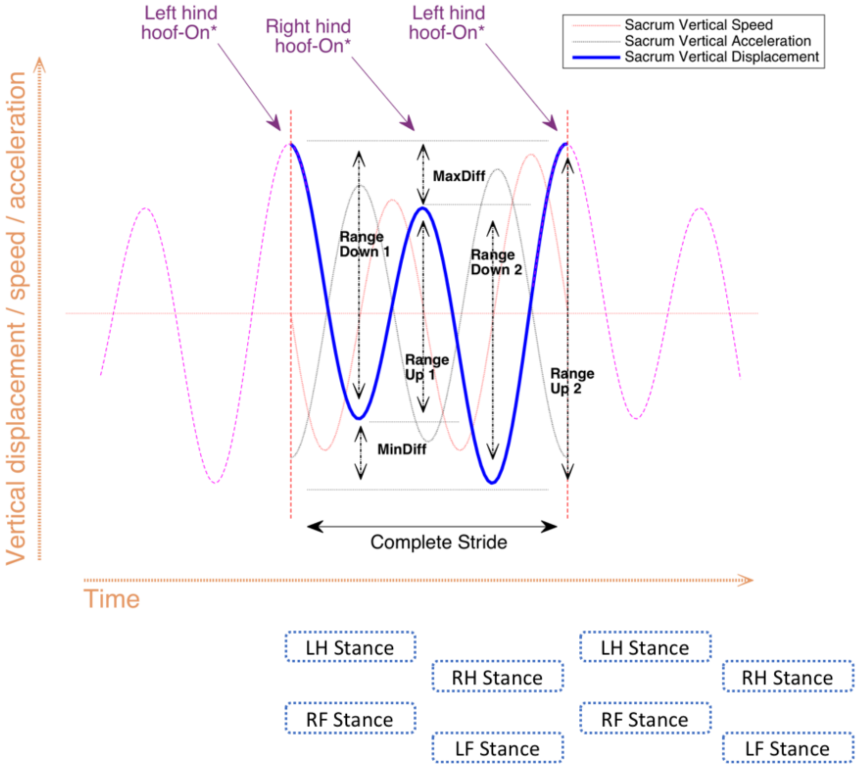

Supplement: Supplementary file 3 — Supplementary Item 3: Visualisation of the symmetry parameters. [file EVJ-51-831-s003.pdf]
