# Supplementary material for: Variation in gait parameters used for objective lameness assessment in sound horses at the trot on the straight line and the lunge
Source: Equine Vet J. 2019 Feb 12;51(6):831–9. doi: 10.1111/evj.13075 (PMC6850282; doi:10.1111/evj.13075)
Supplement: Supplementary file 4 — Supplementary Item 4: Baseline asymmetry (M1) of each horse. [file EVJ-51-831-s004.pdf]

BEVA Equine Veterinary Journal

Supplementary Item 4: Baseline asymmetry mean (s.d.) of each horse on the straight line soft surface (M1).

ID	Head				Withers			
	Maxdiff (mm)	Mindifff (mm)	RUD (mm)	RDD (mm)	Maxdiff (mm)	Mindiff (mm)	RUD (mm)	RDD (mm)
1	-15.5 (31.5)	-9.0(24.9)	-25,2(42.9)	6.5(37.0	15.8(12.4)	5.8 (22.7)	21.6(14.3)	-10.0(33.6)
2	56.1 (47.3)	-0.1(43.8)	50.3(63.6)	-56.2(63.5)	0.2(19.0)	0.7(24.3)	1.1(15.3)	0.5(41.2)
3	25.5 (28.5)	3.7(34.0)	28.0(49.0)	-21.7(40.2)	2.3(16.8)	3.0(24.1)	5.4(11.7)	0.7(40.0)
4	-5.1 (31.4)	-14.1(33.4)	-17.8(47.8)	-9.1(40.7)	0.0(10.8)	-2.0(16.4)	-2.0(13.8)	-2.0(24.4)
5	0.3 (47.6)	-21.5(59.2)	-21.7(77.0)	-21.7(64.2)	-1.1(10.1)	-1.6(18.7)	-2.4(17.6)	-0.5(24.6)
6	-2.2 (31.7)	-10.4(36.6)	-13.0(56.4)	-8.3(35.6)	2.7(10.8)	0.8(23.3)	3.5(18.5)	-1.9(31.5)
7	-27.2 (35.4)	-14.9(42.2)	-39.6(62.1)	12.2(43.3)	0.6(14.4)	-1.7(15.2)	-1.1(11.5)	-2.3(27.5)
8	4.3 (32.4)	-5.1(35.4)	-0.3(46.5)	-9.5(46.1)	1.0(17.4)	0.0(22.3)	-0.8(12.4)	-1.1(38.3)
9	15.4 (23.5)	0.0(46.0)	12.2(45.1)	-15.4(56.5)	5.3(11.0)	6.5(33.9)	11.9(26.2)	1.2(43.0)
10	14.6 (31.9)	16.5(24.7)	31.7(44.4)	1.8(33.9)	15.9(21.2)	10.1(33.7)	26.1(15.6)	-5.8(54.0)
11	20.8 (43.4)	13.2(44.9)	32.9(59.6)	-7.6(54.2)	2.1(14.7)	-4.2(18.9)	-2.0(13.7)	-6.3(31.3)
12	19.5 (27.2)	14.4(39.1)	28.9(50.5)	-5.1(38.6)	-4.4(9.5)	-2.4(20.9)	-6.8(14.7)	2.0(29.1)

ID	Pelvis					
	Maxdiff (mm)	Mindiff (mm)	RUD (mm)	RDD (mm)	HipHikeDiff (mm)	HipDropDiff (mm)
1	-27.3(12.4)	-1.95(17.1)	-29.0(13.0)	25.4(26.7)	-26.4(12.1)	56.8(10.5
2	4.1(11.3)	-5.9(10.2)	-1.7(18.0)	-10.0(11.9)	1.4(14.1)	37.2(18.3)
3	7.6(13.4)	1.7(18.5)	9.7(12.7)	-5.9(29.7)	7.0(10.9)	19.3(11.4)
4	-0.9(12.4)	1.8(27.9)	1.1(22.2)	2.8(37.3)	0.5(14.8)	13.8(10.1)
5	9.5(12.7)	0.4(36.1)	9.9(30.7)	-9.1(44.7)	14.5(19.7)	37.1(13.8)
6	7.2(18.0)	6.6(9.7)	13.8(19.8)	-0.6(21.9)	12.2(23.8)	20.9(14.0)
7	-1.8(13.4)	-3.3(26.4)	-5.2(21.2)	-1.5(36.1)	-4.0(15.2)	30.1(12.4)
8	-5.2(8.0)	-4.2(26.0)	-9.3(26.6)	1.0(28.1)	-5.0(21.3)	29.1(10.5)
9	11.1(7.6)	-8.1(8.5)	3.2(12.7)	-19.2(10.2)	-3.8(12.7)	7.3(12.4)
10	-9.1(8.7)	-9.1(7.3)	-17.9(11.2)	0.0(11.1)	-20.5(13.8)	42.1(12.0)
11	2.3(8.9)	-0.1(10.4)	2.2(15.2)	-2.4(12.3	2.0(13.6)	24.9(10.8)
12	-22.4(8.3)	-3.9(12:9	-26.1(12.4)	18.6(18.0)	-22.5(11.2)	42.7(8.8)
