# Supplementary material for: Variation in gait parameters used for objective lameness assessment in sound horses at the trot on the straight line and the lunge
Source: Equine Vet J. 2019 Feb 12;51(6):831–9. doi: 10.1111/evj.13075 (PMC6850282; doi:10.1111/evj.13075)
Supplement: Supplementary file 5 — Supplementary Item 5: Model estimates square root transformed for the between measurement ‘absolute deviation’. [file EVJ-51-831-s005.pdf]

BEVA Equine Veterinary Journal

Supplementary Item 5: Model estimates (square root transformed) for the measurements 'absolute between day variation'. * Intercept = referenced level (day one, straight line, soft surface).

Square root domain

Head

	MinDiff		MaxDiff		RUD		RDD	
	Estimate	p-value	Estimate	p-value	Estimate	p-value	Estimate	p-value
Intercept*	2.91		2.63		3.48		3.29	
Repetition 2	0.01	0.9	0.06	0.3	0.03	0.6	0.08	0.2
Repetition 3	-0.15	0.04	-0.07	0.3	-0.07	0.4	-0.05	0.5
Repetition 4	-0.18	0.01	-0.05	0.4	-0.12	0.1	-0.12	0.08
Repetition 5	-0.15	0.03	-0.05	0.4	-0.16	0.03	-0.11	0.1
Surface Hard	-0.22	<0.001	-0.14	0.01	-0.12	0.07	-0.36	<0.001
Circle left	0.26	<0.001	0.31	<0.001	0.40	<0.001	0.38	<0.001
Circle right	0.25	<0.001	0.26	<0.001	0.30	<0.001	0.37	<0.001
Day 2	0.02	0.7	-0.04	0.4	-0.05	0.3	-0.02	0.7
Day3	-0.13	0.08	-0.09	0.2	-0.15	0.07	-0.14	0.06

Withers

	MinDiff		MaxDiff		RUD		RDD	
	Estimate	p-value	Estimate	p-value	Estimate	p-value	Estimate	p-value
Intercept*	1.64		1.54		2.05		2.04	
Repetition 2	-0.01	0.7	-0.02	0.4	-0.02	0.5	-0.04	0.3
Repetition 3	-0.05	0.1	-0.05	0.09	-0.05	0.2	-0.05	0.2
Repetition 4	-0.08	0.01	-0.08	0.004	-0.10	0.01	-0.10	0.004
Repetition 5	-0.06	0.03	-0.07	0.01	-0.11	0.001	-0.08	0.02
Surface Hard	-0.15	<0.001	-0.11	<0.001	-0.12	<0.001	-0.19	<0.001
Circle left	0.19	<0.001	0.20	<0.001	0.16	<0.001	0.31	<0.001
Circle right	0.15	<0.001	0.11	<0.001	0.12	<0.001	0.21	<0.001
Day 2	-0.08	<0.001	-0.04	0.02	-0.07	0.004	-0.11	<0.001
Day3	-0.06	0.07	-0.04	0.2	-0.04	0.2	-0.11	0.002

Pelvis

	MinDiff		MaxDiff		RUD		RDD	
	Estimate	p-value	Estimate	p-value	Estimate	p-value	Estimate	p-value
Intercept*	1.83		1.62		2.26		2.18	
Repetition 2	-0.04	0.09	-0.02	0.4	-0.04	0.2	-0.01	0.8
Repetition 3	-0.02	0.5	-0.08	0.01	-0.10	0.01	-0.06	0.09
Repetition 4	-0.07	0.01	-0.07	0.01	-0.08	0.03	-0.09	0.01
Repetition 5	-0.08	0.01	-0.06	0.04	-0.11	0.002	-0.07	0.06
Surface Hard	-0.19	<0.001	-0.07	0.004	-0.22	<0.001	-0.17	<0.001
Circle left	0.10	<0.001	0.16	<0.001	0.11	<0.001	0.15	<0.001
Circle right	0.06	0.01	0.10	<0.001	0.06	0.06	0.12	<0.001
Day 2	-0.06	0.002	-0.03	0.1	-0.07	0.01	-0.03	0.1
Day3	-0.11	<0.001	-0.11	<0.001	-0.14	<0.001	-0.17	<0.001

Hip

	HHDsw		HHDst	
	Estimate	p-value	Estimate	p-value
Intercept*	2.27		2.31	
Repetition 2	-0.02	0.6	-0.03	0.4
Repetition 3	-0.06	0.1	-0.10	0.02
Repetition 4	-0.06	0.1	-0.06	0.1
Repetition 5	-0.12	0.01	-0.17	<0.001
Surface Hard	-0.22	<0.001	-0.19	<0.001
Circle left	0.18	<0.001	0.14	<0.001
Circle right	0.10	0.01	0.05	0.1
Day 2	-0.08	0.01	-0.06	0.04
Day3	-0.15	0.002	-0.10	0.03
