# Supplementary material for: Variation in gait parameters used for objective lameness assessment in sound horses at the trot on the straight line and the lunge
Source: Equine Vet J. 2019 Feb 12;51(6):831–9. doi: 10.1111/evj.13075 (PMC6850282; doi:10.1111/evj.13075)

BEVA Equine Veterinary Journal

**Supplementary Item 6:** Between-measurement variation (Non offset data) per horse and per path over all measurements. Here for the parameters MinDiff head, MinDiff withers and MinDiff pelvis. These data enable the evaluation of the differences between and within horses (relatively small individual boxes compared to the more substantial difference between the different boxplots).

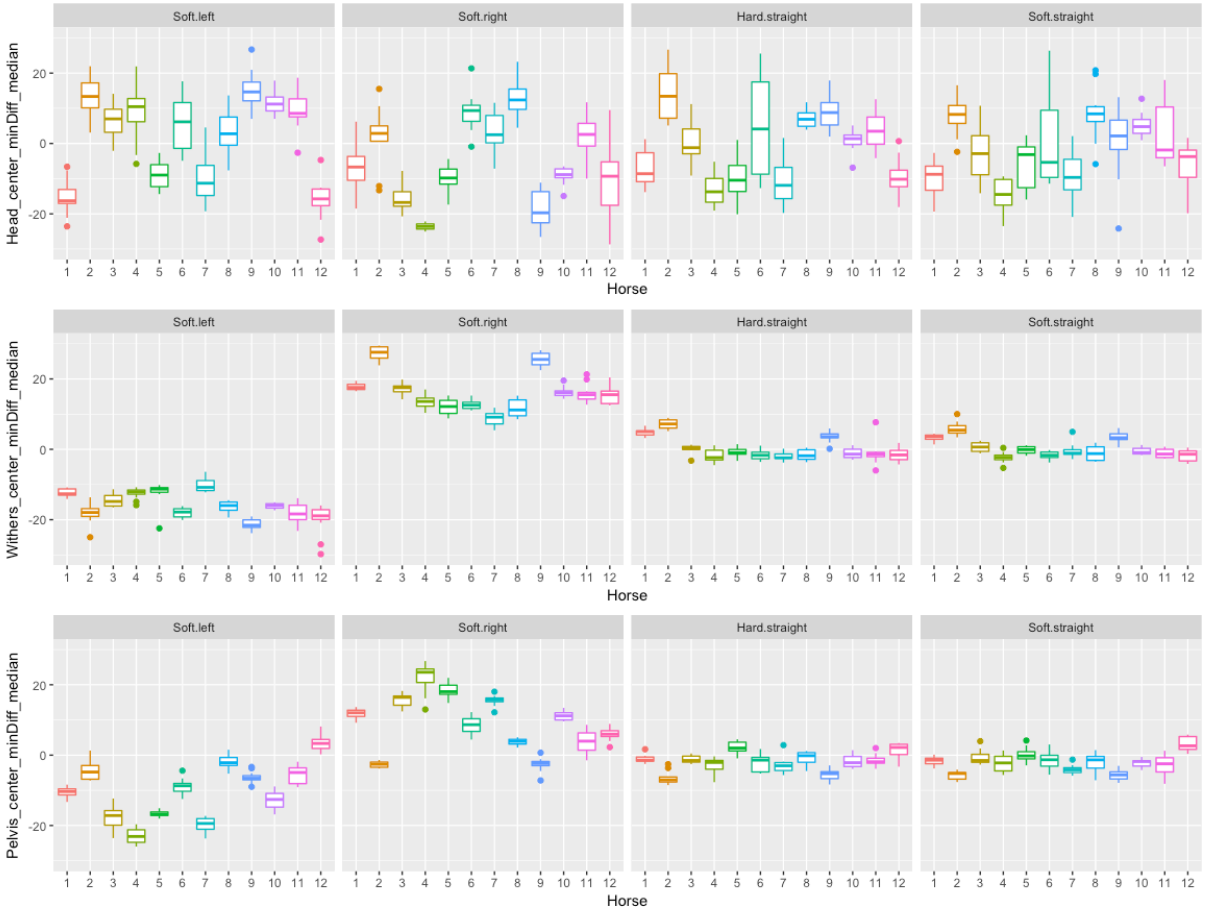

Supplement: Supplementary file 6 — Supplementary Item 6: Between‐measurement‐variation (non offset data) for MinDiff head, withers and pelvis. [file EVJ-51-831-s006.pdf]
